# Validation of the diabetes screening tools proposed by the American Diabetes Association in an aging Chinese population

**DOI:** 10.1371/journal.pone.0184840

**Published:** 2017-09-14

**Authors:** Yu Cho Woo, Chi Ho Lee, Carol H. Y. Fong, Annette W. K. Tso, Bernard M. Y. Cheung, Karen S. L. Lam

**Affiliations:** 1 Department of Medicine, The University of Hong Kong, Hong Kong, Hong Kong SAR; 2 Research Centre of Heart, Brain, Hormone and Healthy Aging, The University of Hong Kong, Hong Kong, Hong Kong SAR; Shanghai Diabetes Institute, CHINA

## Abstract

**Aim:**

Diabetes is a serious global health problem. A simple and effective screening tool should have substantial public health benefit. We investigated the performance of the latest American Diabetes Association diabetes screening methods in our aging Chinese population.

**Methods:**

Subjects without diabetes who returned for the 4th Hong Kong Cardiovascular Risk Factors Prevalence Study in 2010–2012 were evaluated for the probability of having diabetes with reference to the age- and body mass index-based screening criteria (screening criteria) and the diabetes risk test (risk test), and the conclusion drawn was compared to their measured glycaemic status. Diabetes was defined by fasting glucose ≥ 7 mmol/L or 2-hour post oral glucose tolerance test glucose ≥ 11.1 mmol/L.

**Results:**

1415 subjects, aged 58.1±10.2, were evaluated. 95 (6.7%) had diabetes. The risk test showed good accuracy (area under the receiver operating curve 0.725) in screening for diabetes with an optimal cut-off score of five. Compared to the screening criteria, the risk test had significantly better specificity (0.57 vs. 0.41, p<0.001), positive predictive value (0.12 vs. 0.09, p<0.001) and positive diagnostic likelihood ratio (1.85 vs. 1.37, p<0.001). To diagnose one case of diabetes, fewer subjects (11 vs. 18) needed to be tested for blood glucose if the risk test was adopted.

**Conclusion:**

The risk test appears to be a more effective screening tool in our population. It is simple to use and can be adopted as a public health strategy for identifying people with undiagnosed diabetes for early intervention.

## Introduction

Diabetes is a serious global health problem, with the worldwide prevalence of diabetes having more than doubled in the past two decades [1]. Many of these subjects with diabetes, 60.6% in China [2] and 45.8% in the world [3], were undiagnosed, likely attributable to the prolonged asymptomatic phase between the onset of hyperglycemia and diagnosis of type 2 diabetes. Thus at the time of diagnosis, many patients with diabetes may have already developed chronic diabetic complications. In the UK Prospective Diabetes Study, for instance, the prevalence of retinopathy, proteinuria and impaired vibration perception was 36, 1.9 and 11.5% respectively in newly diagnosed patients [4]. In Hong Kong, the prevalence of retinopathy in those with newly diagnosed diabetes was reported to be 22% [5]. Early diagnosis, before the appearance of irreversible tissue damage, offers the best chance for complication prevention. Delivering diabetes screening to all adults in a populous country like China is difficult. However, opportunistic diabetes screening by a practical and effective screening tool, might have substantial public health benefit.

The list of diabetes risk factors is lengthy. It is important that the general public, healthcare professionals and policy makers are informed of the relative strength and the way to make use of the individual risk factors. Screening for diabetes through an assessment of risk factors with regard to an age- and body mass index (BMI)-based criteria (screening criteria); or with the American Diabetes Association (ADA) diabetes risk test (risk test) is recommended by the ADA to guide healthcare providers on whether or not a diagnostic test, i.e. blood glucose or HbA1c measurement, is necessary. The screening criteria suggested that adults aged 45 and older should be tested for diabetes every three years and testing should start earlier than 45 in overweight individuals with one or more of the risk factors for diabetes [6]. In Hong Kong, as 47.0% of our aging population were aged 45 or older in 2015 [7], a large proportion of our population would need regular diagnostic tests for diabetes if we adopt the screening criteria to test all subjects aged 45 or over [6]. A similar problem is anticipated in China, in particular in the urban areas, where population aging is evident, and regular blood glucose screening in all individuals older than 45 years would impose a huge burden to the public healthcare system. The ADA diabetes risk test, which was modified from the model developed using the data of NHANES 1999–2004 by Bang et al in 2009 [8], has been recommended as an additional option for screening [6]. We are interested to know which screening strategy better suits our aging Chinese population.

Here we evaluated the screening criteria and risk test using data from 1415 Hong Kong Chinese subjects who had no diabetes at prior assessments and returned for the 4^th^ Hong Kong Cardiovascular Risk Factors Prevalence Study (CRISPS) in 2010–2012. Our study provides insight on the performance of the two well validated, easily accessible and commonly used ADA diabetes screening recommendations, which involve non-invasive and easily measurable parameters, for diabetes screening in the Chinese population.

## Methods

### Participants

#### CRISPS

Subjects were recruited from the CRISPS, a long-term, population-based, prospective study on the development of cardiovascular risk factors in Hong Kong. In 1995–1996 (CRISPS1), 2,895 unrelated Chinese subjects were invited randomly by their telephone numbers to undergo a detailed assessment [9]. Subjects were contacted for reassessment visits in 2000–2004 (CRISPS2), 2005–2008 (CRISPS3) and 2010–2012 (CRISPS4). Details of medical history taking, anthropometric and biochemical parameters measurements were described elsewhere [10, 11]. The presence of hypertension was defined as blood pressure ≥ 140/90 mmHg or receiving regular antihypertensive treatment [12]. A 75g OGTT was done in all subjects not taking antidiabetic medications when they attended assessment from CRISPS1 to CRISPS4. Diabetes cases were defined as being on medications for diabetes or having diabetes according to the World Health Organization (WHO) 1998 criteria: fasting glucose (FG) ≥ 7 mmol/L or 2hours post OGTT glucose (2-hG) ≥ 11.1 mmol/L.

All subjects with no diabetes at prior assessments (550 or 38.9% were known to have impaired glucose tolerance and/or impaired fasting glucose) who returned for follow-up at CRISPS4 were evaluated for the probability of having diabetes with reference to the ADA screening criteria and risk test, and the conclusion drawn was compared to their measured glycaemic status at the CRISPS4 assessment (S1 File). The ADA diabetes risk test is the risk score based on seven parameters including age, gender, family history of diabetes, history of gestational diabetes in women, history of hypertension, physical activity and body mass index of the individual. Subjects with a score of five or above are considered to have high risk of having diabetes [6]. The study protocol was approved by the Ethics Committee of the Faculty of Medicine, University of Hong Kong. Written informed consent was obtained from all subjects.

#### Statistical analysis

All analyses were performed with SPSS version 23 software (SPSS, Inc., Chicago, IL). Results were presented as mean ± SD or median with interquartile range (IQR) as appropriate. For data that were not normally distributed, natural logarithmic transformation was applied before analyses. In univariate analyses, variables were compared between groups by one-way ANOVA for continuous data and Chi-square test for categorical data as appropriate. The area under the receiver operating characteristic (ROC) curve (AUROC) of the risk score was calculated to assess the performance in identification of diabetes subjects. The optimal cut-off for the risk score was determined by Youden’s index [13] and compared to the ADA recommended cut-off value. The sensitivity, specificity, positive (PPV) and negative predictive values (NPV), diagnostic positive and negative likelihood ratios (DLRs) of the screening criteria and risk test were calculated for comparison. The number needed to test for blood glucose levels to diagnose one case of diabetes (NNT) was calculated by a reciprocal of absolute risk reduction [14]. Comparison of binary diagnostic tests in a paired study design were performed using R (package DTComPair) [15]. Differences in sensitivities and specificities were compared using McNemar’s test [16]. Positive (PPV) and negative predictive value (NPV) were compared as proposed by Moskowitz and Pepe [17], and diagnostic positive and negative DLRs were compared using a regression model approach [18].

## Results

At CRISPS4, 1415 subjects (age: 58.1±10.2) with no diabetes at the last preceding assessment returned for follow up and 95 (6.7%) were diagnosed to have diabetes (S1 File). Table 1 shows that at CRISPS4, compared to those without diabetes, subjects with newly diagnosed diabetes were significantly older, more obese, had greater BMI and waist circumference (WC), and had higher systolic and diastolic blood pressures. They were also more likely to have hypertension (all p<0.001).

**Table 1:** Baseline characteristics of 1415 subjects at CRISPS4.

Variables	All	DM	Non-DM	p-value
Number	1415	95	1320	--
Age, years	58.1±10.2	62.5±10.4	57.8±10.1	**<0.001**
Gender, % men	45.7	46.3	45.6	0.893
Smoking (%)				0.152
Never smoke	72.7	69.5	72.9	
Former smoker	17.2	14.7	17.4	
Current smoker	10.1	15.8	9.7	
Physical inactivity, %	49.2	50.5	49.1	0.787
FG, mmol/L	5.08±0.92	6.89±2.44	4.95±0.48	**<0.001**
2hG, mmol/L	6.63±2.86	14.2±3.89	6.09±1.80	**<0.001**
A1C, %	5.89±0.63	7.04±1.52	5.80±0.40	**<0.001**
A1C, mmol/mol	40.9±6.93	53.4±16.6	39.9±4.42	**<0.001**
First degree of relative with DM, %	28.7	41.1	27.8	**0.006**
History of GDM, %	1.1	2.1	1.0	0.303
BMI, kg/m^2^	24.1±3.47	26.4±4.10	24.0±3.36	**<0.001**
Waist circumference, cm	82.1±9.61	88.7±9.94	81.6±9.41	**<0.001**
Central obesity, %	36.1	63.2	34.2	**<0.001**
Waist-to-hip ratio	0.87±0.07	0.92±0.06	0.87±0.07	**<0.001**
SBP, mmHg	125±18.5	134±18.7	124±18.3	**<0.001**
DBP, mmHg	74.1±10.3	77.6±12.4	73.9±10.1	**0.001**
HT, %	39.1	67.4	37.1	**<0.001**
Triglycerides *, mmol/L	1.10 (0.80–1.50)	1.50 (1.10–2.00)	1.10 (0.80–1.50)	**<0.001**
HDL- Cholesterol, mmol/L	1.49±0.41	1.31±0.34	1.51±0.41	**<0.001**
LDL- Cholesterol, mmol/L	3.12±0.82	3.28±0.89	3.11±0.81	0.055

Data presented as mean±SD or median (interquartile range);

*log-transformed before analysis. Central obesity: WC ≥90 for men and 80 for women; HT, hypertension: BP ≥ 140 / 90mmHg or taking antihypertensives.

The AUROC of the risk test for identification of subject with diabetes was 0.725 with cut-off score being optimal at five, which was identical to the suggested cut-off by ADA (Table 2). The statistical measures of the performance of the two screening methods in evaluation for the subjects at CRISPS4 are shown in Table 3. The risk test had significantly better specificity (0.57 vs. 0.41, p<0.001), positive predictive value (0.12 vs. 0.09, p<0.001) and positive diagnostic likelihood ratio (1.85 vs. 1.37, p<0.001). There was no significant difference in sensitivity, negative predictive value and negative diagnostic likelihood ratios, when compared with the screening criteria (Table 3). In addition, the risk test had smaller NNT (11 vs. 18), compared to the screening criteria. 859 (60.7%) of the CRISPS4 subjects needed to have glucose measurement using the screening criteria but the number would reduce to 647 (45.7%) if the risk test was adopted as the screening tool.

**Table 2:** Different cut-off points for the ADA diabetes risk test when applied in the CRISPS population (n = 1415).

AUROC (95% CI)	Risk score Cut-off	Sensitivity, %	Specificity, %	PPV, %	NPV, %
**0.725 (0.673–0.776)**	1	100.00	0.00	6.7	0.0
	2	100.00	2.0	6.8	100.0
	3	98.9	9.9	7.3	99.2
	4	90.5	28.2	8.3	97.6
	5*	80.0	56.7	11.7	97.5
	6	53.7	78.6	15.3	95.9
	7	20.0	94.2	19.8	94.2
	8	3.2	99.2	21.4	93.4
	9+	0.00	100.00	0.0	93.3

*Optimal cut-off for DM by Youden j index. PPV, Positive Predictive Value; NPV, Negative Predictive Value

**Table 3:** Statistics measures of the performance of the two ADA screening strategies.

Diagnostic test statistic	ADA diabetes risk test Cut-off ≥5	ADA screening criteria	Difference between two tests	Ratio of two tests	p-value
Sensitivity	0.80 (0.72–0.88)	0.81 (0.73–0.89)	-0.01 (-0.11–0.09)		0.8348
Specificity	0.57 (0.54–0.59)	0.41 (0.38–0.43)	0.16 (0.13–0.19)		**<0.00001**
PPV	0.12 (0.09–0.14)	0.09 (0.07–0.11)		1.31 (1.16–1.48)	**<0.00001**
NPV	0.98 (0.96–0.99)	0.97 (0.95–0.98)		1.01 (0.99–1.02)	0.3050
PDLR	1.85 (1.64–2.08)	1.37 (1.23–1.52)		1.35 (1.18–1.55)	**<0.00001**
NDLR	0.35 (0.24–0.53)	0.47 (0.31–0.71)		0.76 (0.45–1.27)	0.2895
NNT	11 (8.3–15.2)	18 (12.3–30.16)			

PPV, Positive Predictive Value; NPV, Negative Predictive Value; PDLR, Positive Diagnostic Likelihood Ratio; NDLR, Negative Diagnostic Likelihood Ratio; NNT, number needed to test for blood glucose levels to diagnose one case of diabetes

## Discussion

In this study, we showed that the suggested recommendations by ADA were effective in screening for undiagnosed cases of diabetes in our population. The ADA diabetes risk test has a higher specificity, positive predictive value and positive likelihood ratio, but lower NNT comparing to the screening criteria with similar sensitivity, NPV and NDRL. The risk test appears to be attractive as a non-invasive means to be used in the Chinese population as this approach has high NPV, which is important as diabetes can be ruled out with high confidence, and the NNT is low.

Both strategies include common conventional risk factors for diabetes but the number of risk factors involved and their application are different. The risk test is based on the scores calculated from seven health-related questions. The performance of its original model has been robustly validated in different populations [8]. While the screening criteria are based mainly on age and BMI as the perquisite factors for prediction, these two risk factors also have much contribution to the scores of the risk test. Despite the mean BMI of our participants being only 24, about 60% of the participants were overweight or obese (Table 1) if we adopted the Asian BMI cut-off for overweight at 23 kg/m^2^ [19, 20] as recommended by the ADA [6]. The scoring of BMI for the on-line version of the risk test has also adopted the Asian criteria. Waist circumference, despite also being used as a predictor in other screening tools [21, 22], was not included in either screening methods recommended by ADA. This may have potential advantage as the measurement of body weight and height is more precise than waist circumference, which is heavily influenced by the anatomic location of measurement [23]. Age is well known to be an important risk factor for diabetes. The screening criteria suggests that even without the presence of other risk factors, regular testing of diabetes should start from the age of 45 and repeat every three years if the previous testing result is negative. If the ADA-suggested age cut point at 45 years is used, for aging populations like the one in CRISPS4, a very high proportion of the population would require regular testing for diabetes. On the other hand, the UK National Screening Committee does not currently recommend universal screening for diabetes based on age, but considers selective screening as part of an overall vascular risk assessment using risk factors as the first stage of selection, followed by the measurement of blood glucose [24]. Our findings would suggest that the ADA diabetes risk test may be considered as a better alternative to the ADA screening criteria in our aging population as fewer people would require regular testing for diabetes but the detection rate would be comparable.

Other health related questions asked in the risk test also include sex, history of gestational diabetes in woman, physical inactivity, family history of diabetes and history of hypertension, which are conventional risk factors for diabetes without involvement of measurements or invasive tests. The former three risk factors, however, did not show statistically significant difference in the diabetes subjects when compared with those without diabetes in our population (Table 1). Each of these three risk factors, however, accounted only for one score point, which was significantly less than the score points related to age and BMI. This might explain why the risk test still performed well despite the inclusion of risk factors which were not statistically different between the diabetes and non-diabetes groups.

The definition of diabetes used in development of the ADA diabetes risk test was based on fasting plasma glucose value. In this CRISPS cohort, subjects were also considered to have diabetes if they fulfilled either the fasting glucose or 2-hG criteria. We did not use HbA1c as a diagnostic criterion for diabetes because the diagnostic criteria have changed over time with HbA1c being adopted from 2011 onwards [25]. The HbA1c criterion would have diagnosed an additional number of subjects with diabetes on top of the glucose criteria at CRISPS3 which might affect the number of people without diabetes at CRISPS4, i.e. the study time frame of this study [26]. Although currently HbA1c measurement may not be available throughout the world, it will be increasingly used clinically for confirming the diagnosis of diabetes, being a conveniently assessable parameter. If we included the HbA1c criterion for diagnosis of diabetes in our analysis, more diabetes cases (157, 11.1%) would be diagnosed in the cohort but, nonetheless, a similar conclusion would be drawn regarding the relative performance of the two screening tests.

The risk test also has the advantage of being simple and applicable in various community or clinical settings. It can be quickly calculated even manually. The time required is minimal and the use of calculator or computer is not essential. Apart from being user-friendly, the risk test has the advantage of better accuracy than existing scores from various populations with an AUROC of 0.79 from the original 6 risk factors model [8]. When applied in the CRISPS4 population, the risk test still maintained reasonably good accuracy with an AUROC of 0.725. This paper is, to our knowledge, the first paper to evaluate this risk test in a homogenous urban Chinese population. Our findings suggest that the well validated ADA diabetes test also has a good validity in detecting Chinese adults with undiagnosed diabetes in our population and could be considered as an option for screening of diabetes in Chinese.

In interpreting our findings, we took into account several limitations. First, at CRISPS4, the average age of the studied subjects was 58, with 43.9% of the subjects aged 45 or above (Table 1). This was not representative of the general population but would be representative of an older population likely to be offered diabetes screening. Second, in a long-term study, there are inevitable losses to follow-up, including deaths. Comparing the baseline characteristics at CRISPS1, the missing subjects were older (49±14 vs. 43±11, P<0.001), more of them were men (51.8% vs. 46.2%, P = 0.003), ever smokers (30.5% vs. 21.7%, P<0.001) and with hypertension (24.6% vs. 13.0% P<0.001). Fewer of them had family history of diabetes (15.2% vs. 18.3%, P = 0.026) The cohort might become less representative of the general population as the defaulted subjects were more likely to be the high risk cases. Our study however has the strengths of being a long-term cohort study in a genetically homogenous Chinese population. The findings should be of considerable value in diabetes screening in Mainland China with its huge aging population.

## Conclusions

In conclusion, we recommend using the ADA diabetes risk test for identification of individuals with increased risk of diabetes in the Chinese population. The test involves only personal medical information and simple non-invasive measurements which should be acceptable by healthcare providers as well as individuals with different education levels. It can also be easily adopted as a public health policy for identifying people with undiagnosed diabetes for early and appropriate treatment to prevent the long-term diabetic complications [27]. Whether this diabetes risk test is more effective than the age-based strategy for diabetes screening is an important public health question to be further investigated in other aging populations.

## Supporting information

S1 File Baseline characteristics and risk of diabetes as assessed by the screening criteria or risk test of 1415 subjects at CRISPS4. Screening criteria, the age- and body mass index-based screening criteria; risk test, the ADA diabetes risk test.
(XLSX)

## References

[1] Zimmet PZ, Magliano DJ, Herman WH, Shaw JE. Diabetes: a 21st century challenge. The lancet Diabetes & endocrinology. 2014;2(1):56–64. doi: 10.1016/S2213-8587(13)70112-8 . 24622669 10.1016/S2213-8587(13)70112-8

[2] Yang W, Lu J, Weng J, Jia W, Ji L, Xiao J, et al Prevalence of diabetes among men and women in China. N Engl J Med. 2010;362(12):1090–101. Epub 2010/03/26. doi: 10.1056/NEJMoa0908292 . 20335585 10.1056/NEJMoa0908292

[3] Beagley J, Guariguata L, Weil C, Motala AA. Global estimates of undiagnosed diabetes in adults. Diabetes Res Clin Pract. 2014;103(2):150–60. doi: 10.1016/j.diabres.2013.11.001 . 24300018 10.1016/j.diabres.2013.11.001

[4] Intensive blood-glucose control with sulphonylureas or insulin compared with conventional treatment and risk of complications in patients with type 2 diabetes (UKPDS 33). UK Prospective Diabetes Study (UKPDS) Group. Lancet. 1998;352(9131):837–53. . 9742976

[5] Wang WQ, Ip TP, Lam KS. Changing prevalence of retinopathy in newly diagnosed non-insulin dependent diabetes mellitus patients in Hong Kong. Diabetes Res Clin Pract. 1998;39(3):185–91. . 9649950 10.1016/s0168-8227(98)00002-3

[6] American Diabetes A. 2. Classification and Diagnosis of Diabetes. Diabetes Care. 2017;40(Suppl 1):S11–S24. doi: 10.2337/dc17-S005 . 27979889 10.2337/dc17-S005

[7] Census and Statistics Department HKSAR. Women and Men in Hong Kong—Key Statistics 2016. http://www.statistics.gov.hk/pub/B11303032016AN16B0100.pdf.

[8] Bang H, Edwards AM, Bomback AS, Ballantyne CM, Brillon D, Callahan MA, et al Development and validation of a patient self-assessment score for diabetes risk. Annals of internal medicine. 2009;151(11):775–83. doi: 10.7326/0003-4819-151-11-200912010-00005 . 19949143 10.1059/0003-4819-151-11-200912010-00005 PMC3633111

[9] Janus ED, Watt NM, Lam KS, Cockram CS, Siu ST, Liu LJ, et al The prevalence of diabetes, association with cardiovascular risk factors and implications of diagnostic criteria (ADA 1997 and WHO 1998) in a 1996 community-based population study in Hong Kong Chinese. Hong Kong Cardiovascular Risk Factor Steering Committee. American Diabetes Association. Diabet Med. 2000;17(10):741–5. . 11110508 10.1046/j.1464-5491.2000.00376.x

[10] Tso AW, Sham PC, Wat NM, Xu A, Cheung BM, Rong R, et al Polymorphisms of the gene encoding adiponectin and glycaemic outcome of Chinese subjects with impaired glucose tolerance: a 5-year follow-up study. Diabetologia. 2006;49(8):1806–15. Epub 2006/06/22. doi: 10.1007/s00125-006-0324-2 . 16788799 10.1007/s00125-006-0324-2

[11] Cheung BM, Wat NM, Man YB, Tam S, Thomas GN, Leung GM, et al Development of diabetes in Chinese with the metabolic syndrome: a 6-year prospective study. Diabetes Care. 2007;30(6):1430–6. doi: 10.2337/dc06-1820 . 17337491 10.2337/dc06-1820

[12] American Diabetes A. 9. Cardiovascular Disease and Risk Management. Diabetes Care. 2017;40(Suppl 1):S75–S87. doi: 10.2337/dc17-S012 . 27979896 10.2337/dc17-S012

[13] DeLong ER, DeLong DM, Clarke-Pearson DL. Comparing the areas under two or more correlated receiver operating characteristic curves: a nonparametric approach. Biometrics. 1988;44(3):837–45. 3203132

[14] Rembold CM. Number needed to screen: development of a statistic for disease screening. BMJ. 1998;317(7154):307–12. . 9685274 10.1136/bmj.317.7154.307 PMC28622

[15] Christian Stock TH. DTComPair: comparison of binary diagnostic tests in a paired study design. R package version 1.0.3. 2014.

[16] Mc NQ. Note on the sampling error of the difference between correlated proportions or percentages. Psychometrika. 1947;12(2):153–7. . 20254758 10.1007/BF02295996

[17] Moskowitz CS, Pepe MS. Comparing the predictive values of diagnostic tests: sample size and analysis for paired study designs. Clinical trials. 2006;3(3):272–9. doi: 10.1191/1740774506cn147oa . 16895044 10.1191/1740774506cn147oa

[18] Gu W, Pepe MS. Estimating the capacity for improvement in risk prediction with a marker. Biostatistics. 2009;10(1):172–86. doi: 10.1093/biostatistics/kxn025 . 18714084 10.1093/biostatistics/kxn025 PMC2639345

[19] Consultation WHOE. Appropriate body-mass index for Asian populations and its implications for policy and intervention strategies. Lancet. 2004;363(9403):157–63. doi: 10.1016/S0140-6736(03)15268-3 . 14726171 10.1016/S0140-6736(03)15268-3

[20] Hsu WC, Araneta MR, Kanaya AM, Chiang JL, Fujimoto W. BMI Cut Points to Identify At-Risk Asian Americans for Type 2 Diabetes Screening. Diabetes Care. 2015;38(1):150–8. doi: 10.2337/dc14-2391 . 25538311 10.2337/dc14-2391 PMC4392932

[21] Lindstrom J, Tuomilehto J. The diabetes risk score: a practical tool to predict type 2 diabetes risk. Diabetes Care. 2003;26(3):725–31. . 12610029 10.2337/diacare.26.3.725

[22] Lee YH, Bang H, Kim HC, Kim HM, Park SW, Kim DJ. A simple screening score for diabetes for the Korean population: development, validation, and comparison with other scores. Diabetes Care. 2012;35(8):1723–30. doi: 10.2337/dc11-2347 . 22688547 10.2337/dc11-2347 PMC3402268

[23] Mason C, Katzmarzyk PT. Variability in waist circumference measurements according to anatomic measurement site. Obesity (Silver Spring). 2009;17(9):1789–95. doi: 10.1038/oby.2009.87 . 19343017 10.1038/oby.2009.87

[24] Waugh NR, Shyangdan D, Taylor-Phillips S, Suri G, Hall B. Screening for type 2 diabetes: a short report for the National Screening Committee. Health technology assessment. 2013;17(35):1–90. doi: 10.3310/hta17350 . 23972041 10.3310/hta17350 PMC4780946

[25] International Expert C. International Expert Committee report on the role of the A1C assay in the diagnosis of diabetes. Diabetes Care. 2009;32(7):1327–34. doi: 10.2337/dc09-9033 . 19502545 10.2337/dc09-9033 PMC2699715

[26] Woo YC, Cheung BM, Yeung CY, Lee CH, Hui EY, Fong CH, et al Cardiometabolic risk profile of participants with prediabetes diagnosed by HbA1c criteria in an urban Hong Kong Chinese population over 40 years of age. Diabet Med. 2015 doi: 10.1111/dme.12691 . 25594838 10.1111/dme.12691

[27] Holman RR, Paul SK, Bethel MA, Matthews DR, Neil HA. 10-year follow-up of intensive glucose control in type 2 diabetes. N Engl J Med. 2008;359(15):1577–89. doi: 10.1056/NEJMoa0806470 . 18784090 10.1056/NEJMoa0806470

